# The Association Between RACK1 Gene Polymorphisms rs1279736 and rs3756585 in Uterine Cervical Cancer with the Risk Factor of HPV: A Focus on Squamous Cell Carcinoma (SCC)

**DOI:** 10.30699/ijp.2024.2019194.3236

**Published:** 2025-01-10

**Authors:** Maryam Fekri Soofi Abadi, Fatemeh Hashemi, Saeedeh Shojaeepour, Sahar Amirpour-Rostami, Mohammad Reza Zangouey, Sara Pourshaikhali, Fatemeh Pouya, Shahriar Dabiri

**Affiliations:** 1 *Pathology and Stem Cell Research Center, Kerman University of Medical Sciences, Kerman, Iran*; 2 *Department of Biochemistry & Molecular Biology, Arnie Charbonneau Cancer Institute, Cumming School of Medicine, University of Calgary, Calgary, AB, Canada*; 3 *Department of Basic Sciences, Faculty of Veterinary Medicine, Shahid Bahonar University of Kerman, Kerman, Iran*; 4 *Pharmaceutics Research Center, Institute of Neuropharmacology, Kerman University of Medical Sciences, Kerman, Iran*; 5 *Department of Anatomy, Afzalipour School of Medicine, Kerman University of Medical Sciences, Kerman, Iran*

**Keywords:** Cervical cancer, Genetic polymorphisms, HPV, RACK1

## Abstract

**Background & Objective:**

Cervical cancer is the second most common malignancy among women worldwide. The Receptor for Activated C Kinase 1 (RACK1) has a key role in regulating the pathways of cell signaling, angiogenesis, cell migration, invasion, and metastasis. This study investigated the association of polymorphisms rs1279736 and rs3756585 with cervical cancer.

**Methods:**

In this study, 100 LB pap-smear and paraffin block samples of cervical squamous carcinoma associated with Human *papillomavirus (HPV)* were selected along with 100 LB smear cytology samples from healthy women with normal pap smears, serving as the control group. Genomic DNA was extracted from the samples, and the polymorphisms rs1279736 and rs3756585 in the RACK1 genes were examined using the RFLP-PCR and ARMS-PCR methods, respectively**.**

**Results:**

The rs1279736 polymorphism shows that the chance of cervical cancer in individuals with CA and AA genotypes is 3.772 and 1.852 times that of CC genotypes, respectively. This suggests that the A allele is involved in developing cervical cancer (*P*=0.005; OR=2.113). The rs3756585 polymorphism indicates that the chance of cervical cancer in TG and GG genotypes is 0.373 and 4.235 times that of TT genotypes, respectively. This also points towards the involvement of the G allele in the development of cervical cancer (*P*=0.032; OR=1.800). Also, there was no significant relationship between the genotype of polymorphisms, age, and subtype of papillomavirus 16 and 18 in patients.

**Conclusion:**

The present study was conducted for the first time. Our results showed that two polymorphisms are significantly associated with cervical cancer.

## Introduction

Cervical cancer is the fourth most common cancer in women globally, with around 660,000 new cases and around 350,000 deaths in 2022 (1, 2). The etiology and pathogenicity of the cervical cancer includes various environmental and genetic factors that lead to epithelial cell transformation (3, 4). Among papillomavirus-related infections and cancers, Human Papillomavirus (HPV) is found in 99% of cervical cancers, making it an essential factor in the development of cervical cancer (5-7). Papillomaviruses are a small group of uncovered viruses with 20-sided capsids and double-stranded DNA that have 8 primary regulatory genes and 2 structural genes of L1 and L2 capsid with delayed expression. These viruses infect squamous epithelial cells in different areas (4, 8-10). Among the various genotypes of papillomavirus, the HPV16 and HPV18 regulatory genes are known to be the most common HPV genotypes, found in more than 70% of cervical cancer specimens worldwide (11-13). HPV is one of the most important oncoviruses that causes lesions not only in the genital area but also in the respiratory tract, lungs, mucous membranes, scalp, neck, and so on. Several factors, including genetic alterations such as polymorphisms and epigenetic modifications, can potentially influence gene expression (14-18).

RACK1 acts as a scaffold for numerous protein kinases, making it integral to various cellular responses, including signal transduction, cell growth, differentiation, and immune responses. Elevated expression of RACK1 has been demonstrated to correlate with the initiation and advancement of numerous malignancies (19). This multifaceted role in cancer progression holds potential significance in the context of cervical cancer. Furthermore, regulating RACK1 gene expression at the transcriptional level, coupled with the discovery of polymorphisms in the RACK1 promoter associated with increased expression (20), adds a genetic dimension to its potential role in cervical cancer, warranting comprehensive exploration.

Traditional cytological methods, such as the Pap smear, face challenges in accurately predicting cervical cancer. These limitations have spurred the development of more sensitive diagnostic techniques, particularly in detecting high-risk HPV genotypes through PCR-based approaches (6, 21, 22). As a result, there is a growing need to introduce new diagnostic methods with high sensitivity, specificity, rapid results, and affordability.

The aim of this study is to investigate RACK1 gene polymorphisms, especially rs3756585 and rs1279736, using ARMS PCR and RLFP PCR methods on HPV-induced cervical cancer samples, focusing on squamous cell carcinoma (SCC) compared to the control group.

## Material and Methods

### Sample Collection

100 LB pap-smear and paraffin blocks, which were diagnosed by H&E staining as squamous cell carcinoma of cervical cancer, were collected from the pathology laboratory of Afzalipour Hospital, Kerman, South-Eastern Iran, spanning the years 2017 to 2021. These samples also included cancers associated with 16,18 subtypes of human papillomavirus (HPV). Additionally, we selected 100 Pap smear cytology samples from healthy women with normal pap smears, confirmed to be HPV-negative by PCR, to serve as the control group. Exclusion criteria included pregnancy, vaginal bleeding, symptomatic cervical or vaginal infections, previous hysterectomy, a history of previous cervical cancer, radiation therapy, and the use of immunosuppressive drugs. This project was approved by the ethical board of Kerman University of Medical Sciences (IR.KMU.REC.1401.219). All the participants filled out the written informed consent before collecting the samples (23).

### Human Papilloma Virus Screening

Liquid-based cytology (LBC) and a liquid-based Pap test (Ilia Tech Kimia Sahand, Iran) were employed to collect cytological control samples. HPV-DNA testing was conducted using the EXTRA II INNO-LiPA HPV genotyping kit from Fuji Rebio Europe N.V. (Belgium), which is based on the principles of reverse hybridization. In this process, a 65 bp region of HPV DNA, specifically the L1 region known as SPF10, was amplified by biotinylated primers. After denaturation, the biotinylated amplicons were hybridized with distinct probes. Specific primers for the human HLA-DPB1 gene were included to assess the quality of the extracted DNA. Subsequently, streptavidin-conjugated alkaline phosphatase was applied, followed by incubation with BCIP/NBT for visual confirmation (23, 24).

### Histological evaluation

The 3-5 micrometer sections were prepared from the paraffin tissues and stained with Hematoxylin and Eosin. The histopathological assessment of the uterine cervical lesion was diagnosed as squamous cell carcinoma done by an experienced anatomical pathologist using a light microscope (Olympus/BX51, Japan).

### DNA Extraction

DNA extraction was performed using the Cinapure DNA kit (CatNo: EX6011) according to the protocol. First, the samples were incubated in prelysis buffer and ribonuclease for 3 hours at 55°C until complete digestion was achieved. Subsequently, lysis solution was added and vortexed for 20 seconds, followed by the addition of precipitation solution. The resulting solution was then transferred to a spin column. After undergoing two washes with wash buffers I and II, the samples were centrifuged at 13,000 rpm for 1 minute. Finally, the samples were incubated with an elution buffer at 65°C for 3-5 minutes. To assess the extracted DNA's quality, quantity, and purity, measurements were performed by determining the optical density at 260 and 280 nm using a Nanodrop ND2000 (Thermo) and through electrophoresis on an agarose gel.

### Primer Design

In this study, primers were developed using the Primer3 Bioinformatic software and subsequently validated by the NCBI through Primer-BLAST. The Korean Bioneer Company synthesized these primers. The primers utilized for the rs1279736 and rs3756585 polymorphisms are detailed in Table 1.

### Identification of rs1279736 Polymorphism by using RFLP-PCR Method

**Restriction Fragment Length Polymorphism**

The polymerase Chain Reaction (RFLP-PCR) method was used to determine rs1279736 polymorphism. The PCR reaction was performed using the primers listed in Table 1 at a final volume of 25 μL, including 10 picomoles per primer, 12.5 μL of Mastermix Ampliqon, and 50 ng of genomic DNA. The thermal program of the reaction is shown in Table 2. First, 14 μL of PCR product was exposed to MSP1 (Thermo Cat No:ER0541). Then, samples were incubated overnight at 37°C for the effect of restriction enzyme and incubated for 20 minutes at 70°C for inactivation of the enzyme. Finally, to view the results, 18 μL of the RFLP reaction product was electrophoresed with Sybersafe (cat no: S33102 Invitrogen) on 3% agarose gel along with 4 μL of 100bp marker. C > A at nucleotide number rs1279736 is not detected by restriction enzyme MSP1 at C^CGG sites. As a result, polymorphism is determined for each sample C (Wild) = 49,182, A (Mutant) = 231 and AC (Heterozygous) = 231‚49‚182.

### Investigation of rs3756585 Polymorphism by ARMS-PCR Method

To detect mutations in the patients' samples, 12.5 μL of PCR mix and 50 ng of template DNA were each added to separate microtubes. Subsequently, forward and rev

erse primers for the normal allele were introduced into the first microtube, while forward and reverse primers for the mutant allele were added to the second microtube. Internal control (IC) primers of lactate dehydrogenase A (LDHA) were then included in both microtubes. The PCR reaction was conducted using a Biorad C1000 thermocycler, following the time-temperature schedule detailed in Table 2. The resulting product had a band size of 231 bp, the resulting product had band sizes of 231 bp and 420 bp (IC), and the optimal annealing temperature was determined through testing with a temperature gradient. 

### Statistical Analysis

Statistical analysis of the data was performed using SPSS version 26 (SPSS Inc., Chicago, Ill., USA) and GraphPad prism8 software was used for drawing diagrams. An alpha error of 5% (95% confidence interval) is considered as a limit for rejecting or confirming the null hypothesis. All mean comparison tests were performed bilaterally. The Chi-Square test was used in this study.

## Results

One hundred patients with cervical cancer caused by papillomavirus diagnosed with SCC and 100 healthy subjects were analyzed for RACK1 polymorphism genotypes by H&E staining. Some individuals were not successfully genotyped for all tested genotypes and were excluded, which was probably due to the poor quality of the samples. This left 80 and 96 people in the patient and control groups, respectively.

### Histopathological Findings

**Fig. 1:**
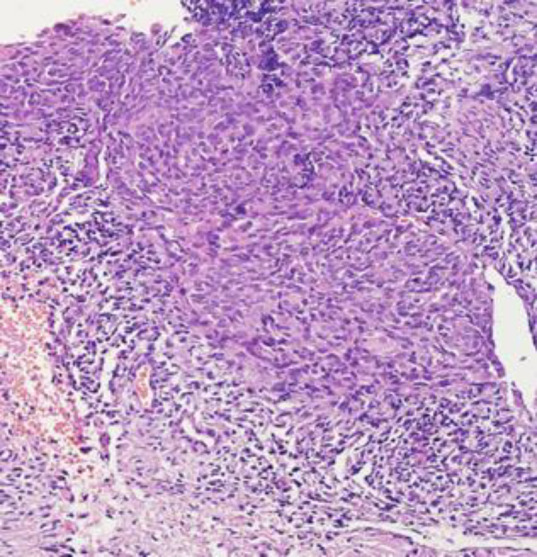
Shows proliferation of malignant squamous cells with moderate nuclear pleomorphism and also infiltrating to the stroma.

**Fig. 2:**
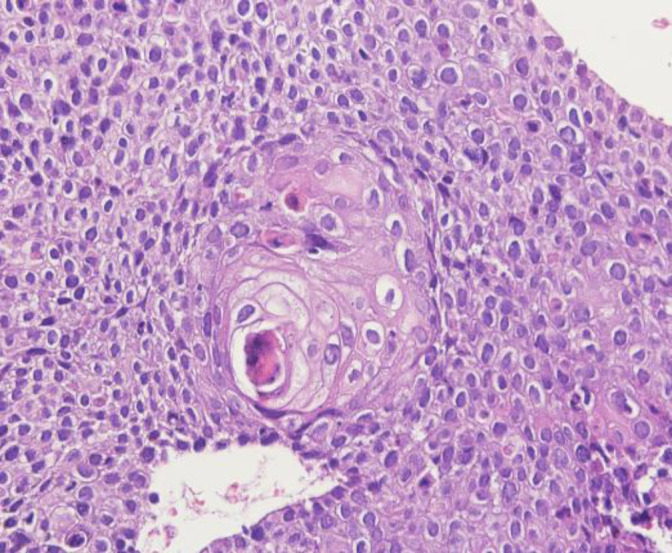
Presence of the occasional koilocytes within the neoplastic keratinocytes.

Most of the cases showed a spectrum of squamous metaplasia, koilocytosis, various grades of dysplasia, carcinoma in situ, and finally malignant proliferation of the squamous cells with nuclear pleomorphism, prominent nucleoli, and multifocal stromal invasion of the uterine cervix. Squamous carcinoma subtypes included large cell noncell keratinizing type 60%, large cell keratinizing type 35%, and small cell type 5% (Fig 1, 2). 

### Relationship Between Genotypes of Rack1 Polymorphisms with Age and HPV Subtypes (16,18) in Patients

The age range of the patient group was between 45-73 (mean age: 55.83±05), and the age of the control group was between 28-68 years (mean age: 48.49±10.41). In this study, the patients had 42.5% subtype 16, 20% subtype 18, and 37.5% both subtype 16/18 of HPV. The findings show that there was no significant correlation between the frequency of genotypes and age and no significant relationship between HPV subtypes (16 and 18) and rs1279736 and rs3756585 polymorphisms. Analysis of the relationship between the two polymorphisms (rs1279736 and rs3756585) and age, as well as HPV type (16, 18), is detailed in Tables 3 and 4.

### Relationship Between Genotypes of Rack1 Polymorphisms with the Risk of Cervical Cancer

In the rs1279736 polymorphism, it was found that the percentage of CC, CA, and AA genotypes was 56.3%, 31.3%, 12.5% for the patient group and 78.1%, 12.5%, 9.4% for the healthy group. CC genotype was considered as a reference. The genotype of rs1279736 polymorphism has a significant relationship with cervical cancer(*P*=0.005). According to the calculated OR number, the relationship of this polymorphism with the condition of disease showed that the chance of disease in people with CA and AA genotypes is 3.472 and 1.852, respectively, compared to CC people. In simpler words, people with genotypes CA and AA get more than those with genotypes CC. Also, the frequency of people with A allele is significantly higher than the C allele (*P*=0.005; OR=2.113), and this suggests that allele A is involved in causing the disease. The changes in the total number of mentioned samples and the frequency of rs1279736 and rs3756585 genotypes in patients with cervical cancer and in the control group are given in Table 5.

In rs3756585 polymorphism, the percentages of TT, TG, and GG genotypes are 71.3%, 7.5%, and 21.3% for sick people and 74.0%, 20.8%, and 5.2% for healthy people, respectively. rs3756585 polymorphism genotype has a significant relationship with cervical cancer, and it is interesting here. This relationship is such that the chance of cervical cancer in TG and GG genotypes is 00.373 and 4.235 times that of TT, respectively. Since the frequency of the G allele in patients was significantly higher (*P*=0.032; OR=1.800), it seems that the G allele is involved in the development of this disease.

After examining whether the allele frequencies conform to the Hardy-Weinberg equilibrium for the rs1279736 and rs3756585 polymorphisms, it was found that the frequencies of its alleles significantly deviated from the Hardy-Weinberg equilibrium (*P*<0.00001). This deviation suggests that allelic frequencies in the population are influenced by factors such as natural selection, migration, or mutation Notably.

### RFLP and ARMS PCR Product Results

**Fig. 3:**
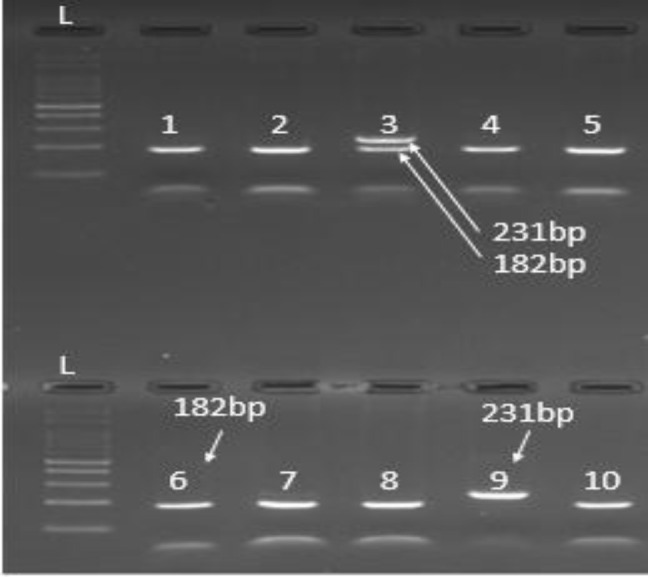
The effect of MSP1 enzyme on the PCR product of rs1279736 polymorphism of rack1 gene. Well 3: heterozygous (AC); Well 1, 2, 4, 5, 6, 7, 8, 10: homozygous wild (CC); Well 9: homozygous mutant (AA) and Well L: 100bp ladder

**Fig. 4:**
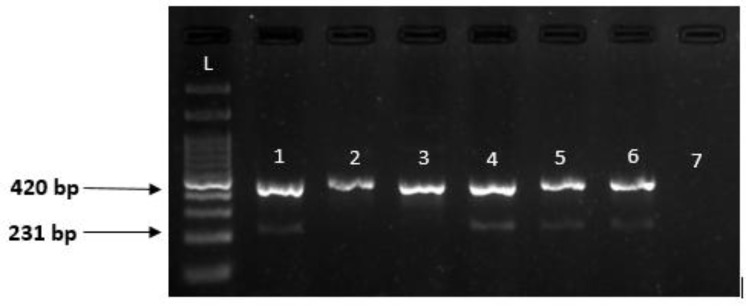
2% agarose gel related to electrophoresis and PCR product of rs3756585 rack1 polymorphism; Well 1,2: show homozygous wild (TT); Well 3,4: homozygous mutant (GG); Well 5,6: heterozygous (TG) and Well 7: negative control; Well L: 100bp ladder

After complete PCR and electrophoresis, staining with a safe Syber dye was performed. The results of RFLP and ARMS are shown in Figures 3 and 4, respectively.

## Discussion

Cervical cancer ranks as the second most prevalent malignancy among women worldwide, with approximately 500,000 new cases reported each year across diverse communities. Notably, the primary driver of cervical carcinoma's development and progression is the human papillomavirus (HPV) (11). The results of typing HPV-positive samples indicate that HPV16 is the most common type isolated from cervical cancer patients. Detection methods, such as regular Pap smears and molecular pathology tests employing PCR and DNA probes, have been crucial in identifying HPV in cervical tissue or swab samples, thus allowing for interventions like freezing, burning, or drug treatments to prevent progression toward cancer (11, 12). The significance of cervical cancer underscores the importance of investigating genotypes, polymorphisms, and genetic variations in diverse populations, considering factors like race and geographical differences. Such studies are invaluable in estimating cancer risk and advancing therapeutic objectives (25-28). Several studies have shown that RACK1 promotes tumor progression and malignancy in colorectal, esophageal, and squamous cell carcinomas of the mouth and lungs, as well as increases the proliferation, migration, and metastasis of breast cancer and is considered an independent prognostic factor (29).

RACK1, a 36 kDa protein containing repeat aspartate-glutamate (WD) domains, is an intracellular receptor for PKCβ II protein and functions as a scaffold for various kinase proteins and receptors. This protein plays a pivotal role in numerous cellular responses, including signal transduction, cell growth, differentiation, and immune responses. One of the important factors regulating the expression of this gene is regulation at the transcriptional level. It has been shown that several polymorphisms in the RACK1 promoter are associated with increased expression, the most important and frequent polymorphisms being rs1279736 and rs3756585, which have a high prevalence. (20, 30, 31). The polymorphisms rs1279736 and rs3756585 are situated within the promoter region of the RACK1 gene, specifically at -283 and -123 bases from the transcription initiation site (20). Their genomic locations are chr5:181244189 and chr5:181244029 in the GRCh38.p14 reference genome. The standard HGVS nomenclature for these polymorphisms is NC_000005.9: g.180671189C>A for rs1279736 and NC_000005.9: g.180671029T>G for rs3756585. It has been observed that the C allele for rs1279736 and the T allele for rs3756585 are the most prevalent in various populations.

RACK1's role in cancer progression is complex and context-dependent, acting as both a tumor suppressor and an oncogene in different types of cancers. Its interactions with various cellular signaling pathways and its impact on specific cancer types highlight its critical role in the initiation and progression of various cancers. (30, 32-34). Studies have shown that RACK1 is upregulated in cervical cancer tissues compared to normal tissues, with its expression level increasing from cervicitis to cervical intraepithelial neoplasia (CIN) to carcinoma. This upregulation of RACK1 is positively correlated with tumor grading, suggesting its involvement in tumorigenesis (35). Hao Wu *et al.* showed that The HPV E6 oncogene has been found to elevate RACK1 levels at the post-translational level in cervical cancer. This upregulation of RACK1 significantly boosts tumor cell invasion and epithelial-mesenchymal transition (EMT), while also facilitating lymph angiogenesis and lymph node metastasis in a manner dependent on galectin-1 (34).

Yi-Young Choi *et al.* performed on non-small cell lung cancer samples, determined that rs1279736C>A and rs3756585T>G polymorphisms in the RACK1 promoter were most associated with survival outcomes. Also, in vitro promoter assay and EMSA showed that rs3756585 T-to-G mutation increased transcription factor binding and RACK1 promoter activity (20). We determined that the genotype of rs1279736 polymorphism has a significant relationship with cervical cancer, and the chance of cervical cancer in CA and AA genotypes is 3.772 and 1.852 times of CC, respectively. And since the frequency of A allele in patients is significantly higher (P-Value=0.005; OR=2.113), it seems that A allele is involved in the development of this disease. The present study also determined that the rs3756585 polymorphism genotype has a significant relationship with cervical cancer; interestingly, this relationship is such that the chance of cervical cancer in TG and GG genotypes is 00.373 and 4.235 times of TT, respectively. This is a rare outcome and is very similar to the high frequency of individuals heterozygous for the sickle red cell mutation that has developed in parts of Africa due to malaria resistance. Since the frequency of G allele in patients was significantly higher (*P*=0.032; OR=1.800), it seems that G allele is involved in the development of this disease. In fact, our current study showed the functional importance of two polymorphisms (rs1279736 and rs3756585) in the RACK1 promoter in cervical cancer. These polymorphisms may act as prognostic factors in cervical cancer. However, due to the lack of more prognostic data from clinical samples, we could not clarify the relationship between them and the genotypes of RACK1gene polymorphism, and the available data about age and the subtype of HPV in patients showed no significant relationship. More studies on RACK1gene polymorphisms are needed to clarify their mechanisms. With the recommendation that research the future includes larger and more diverse populations to account for racial and geographic differences and advance cancer treatment goals.

**Table 1:** Sequence of primers used for PCR (RFLP and ARMS)

**Forward: 5’-TGGAACAGGCGGAGCTCG-3’**	**RFLP** **rs1279736**
**Reverse: 5’-AAAAGGCAGAGGGAAATTGCAA-3’**	
**Forward: 5’-TGGAACAGGCGGAGCTCG-3’**	**ARMS** **rs3756585**
**Reverse (A allele): 5’- AAAAGGCAGAGGGAAATTGCAA-3’**	
**Reverse (C allele): 5’- AAAAGGCAGAGGGAAATTGCAC-3’**	
**Forward: 5’- CACCTCTGACGCACCACTG-3’**	**LDHA**
**Reverse: 5’-CAGGGTTGCCCAAGAATAGC -3’**	

**Table 2:** The temperature program used for PCR (RFLP and ARMS)

	35 cycles				
Final Elongation	Extention	Annealing	Denaturation	Denaturation	
5 min 72^0C^	20 s 72^0C^	20 s 60^0C^	20 s 94^0C^	5 min 94^0C^	RFLP
5 min 72^0C^	20 s 72^0C^	30 s 62^0C^	20 s 94^0C^	5 min 94^0C^	ARMS

**Table 3:** Relationship between genotypes of rs1279736 polymorphism with age and HPV in patients

Clinical features		CC	CA	AA	p-value
Age (in years)	≤55	24 (66.6%)	9(25%)	3(8.3%)	0.224
	>55	21 (47.7%)	16 (36.4%)	7(15.9%)	
Subtype of HPV	16(N=34)	16 (47%)	14(41.1%)	4 (11.8%)	0.188
	18(N=16)	13 (81.3%)	2 (12.5%)	1 (6.25%)	
	N=30))16,18	16(53.3%)	9(30%)	5(16.7%)	

**Table 4:** Relationship between genotypes of rs3756585 polymorphism with age and HPV in patients

Clinical features		TT	TG	GG	p-value
Age (in years)	≤55	31 (63.3%)	4 (8.2%)	14(28.6)	0.111
	>55	26 (83.9%)	2 (6.5%)	3(9.7%)	
Subtype of HPV	16(N=34)	24 (70.5%)	3(8.9%)	7 (20.6%)	0.463
	18(N=16)	14(87.4%)	1 (6.3%)	1 (6.3%)	
	N=30))16,18	19(63.3%)	2(6.7%)	9(30%)	

**Table 5:** Impact of Single Genotypes of RACK1 on the Frequency of The Cervical Cancer

Genotype	Control	Cervical cancer	OR	p-value
rs1279736	(n=96)	(n=80)		
CC	75(78.1%)	45(56.3%)	1 (Reference)	
CA	12(12.5%)	25(31.3%)	3.472	0.005
AA	9(9.4%)	10(12.5%)	1.852	0.005
Allele C	162(84.37%)	115(71.87%)	1	
Allele A	30(15.62%)	45(28.12%)	2.113	0.005
rs3756585	(n=96)	(n=80)		
TT	71(74.0%)	57(71.3%)	1	
TG	20(20.8%)	6(7.5%)	0.373	0.001
GG	5(5.2%)	17(21.3%)	4.235	0.001
Allele T	162(84.37%)	120(75.00%)	1	
Allele G	30(15.62%)	40(25.00%)	1.800	0.032

## Conclusion

Our results show that RACK1 gene polymorphisms rs1279736 and rs3756585 have a significant effect on cervical cancer. The rs1279736 polymorphism showed an increased risk of cervical cancer in individuals with CA and AA genotypes compared to those with CC genotypes, suggesting the involvement of the A allele in the development of cervical cancer. Similarly, the rs3756585 polymorphism showed an increased risk of cervical cancer in subjects with TG and GG genotypes compared to subjects with TT genotype, suggesting the involvement of the G allele in the development of the disease. Since this is the first study in the field of investigating Rack1 polymorphisms, it is hoped that more studies will be conducted in different populations that will lead to the early detection of cervical cancer and its treatment.
